# Bloomingdale Asylum, New York

**Published:** 1849-04-01

**Authors:** 


THE JOURNAL

OF

PSYCHOLOGICAL MEDICINE

AND

MENTAL PATHOLOGY.

APRIL 1, 1849.

### Analytical Reviews.

ART. I.—*History, Description, and Statistics of the Bloomingdale Asylum for the Insane, State of New York, America.* By PLINY EARLE, M.D., Physician to the Institution. New York, 1848.

WE cannot conceive a more interesting record than the history of a long-established and well-conducted asylum for the treatment of the insane. What a field of philosophical inquiry does it not open to the moralist, the physician, and the psychologist? We have been much gratified with the work before us. The Bloomingdale Asylum has, we believe, justly earned for itself great reputation, and deserves to have its history chronicled. Its origin dates as far back as 1791, to the foundation of the New York Hospital; but it was not till 1797 that any insane cases were admitted into the wards of the institution. Previously to 1803, the number of lunatics received into the hospital amounted to 215. It was thought necessary, in consequence of the applications for admission increasing, to erect a spacious stone edifice near the hospital, in the same enclosure. This was finished in 1808. This building, which was termed the "Lunatic Asylum," was placed under the medical care of Dr. Archibald Bruce. Previously to 1811, 643 patients had been received into the asylum; and from 1811 to 1821, the admissions amounted to 1533. During that period the deaths were 154; cures, 704; relieved, 239; discharged by request, 278. The mean or average daily number of patients residing in the asylum, from 1821 to 1828, was 110·44. At the period when this institution was opened in 1821, there were but four other public asylums devoted to the insane in the United States; but before the year 1844, no less than sixteen new asylums were opened, making the whole number in the country twenty-one.

"In 1821, this institution was alone in the State of New York, and there was none in the neighbouring States nearer than that at Hartford, Connecticut, on the one hand, and that at Frankford, near Philadelphia, on the other. The extent of territory from which it might be expected that the Bloomingdale Asylum would receive patients was consequently very large. The establishment of new institutions necessarily tended to restrict its limits. This was the fact, particularly in reference to the New York City Pauper Asylum, opened in 1839, and the New York State Asylum, at Utica, opened in 1843. The former took directly from this asylum, twenty-nine of its inmates, and prevented any future admissions of pauper patients from the city; and the latter, occupying a central position in the State, received from all the inland and western counties patients, at both private and public expense, who would otherwise have been brought to Bloomingdale."

To those who take an interest in the progress of the enterprise for the melioration of the condition of the insane, it cannot fail to be a source of gratification that, as appears by the following statement, the accommodations for this afflicted class are becoming far more nearly adequate to their necessities.

On the 31st of December, 1821, there were eighty-two patients at the Bloomingdale Asylum, the only institution devoted to the curative treatment of insanity, at that time existing in the State of New York.

| | | | PATIENTS. |
|---|---|---|---|
| On 31st Dec. 1844, there were | | at the Bloomingdale Asylum . . . | 104 |
| " | " | at the New York State Asylum . . | 260 |
| " | " | at the New York City Asylum . . | 352 |
| " | " | at Dr. White's, Institution, Hudson . | 20 |
| " | " | at Dr. Macdonald's, New York, City | 260 |
| | | Total . . . . . | 751 |

Thus, in *twenty-three years*, the number of patients in asylums increased from *eighty-two* to *seven hundred and fifty-one*.

In July, 1847, the number of patients in the institutions just mentioned, and in one which was opened since 1844, was as follows:—

| | |
|---|---|
| Bloomingdale Asylum . . . . . . . . . . | 142 |
| New York State . . . . . . . . . . . . | 430 |
| New York City . . . . . . . . . . . . | 417 |
| Queen's County (Flatbush) Asylum . . . . . | 70 |
| Dr. White's . . . . . . . . . . . . . | 20 |
| Dr. Macdonald's . . . . . . . . . . . . | 30 |
| | 1109 |

Thus, more than one thousand one hundred of the insane are now provided for at the institutions within that State; and yet the wants of the community are not fully supplied.

"The Bloomingdale Asylum for the Insane is within the limits of the municipal jurisdiction of the city of New York. It is on 117th street, between the tenth and the eleventh avenues, seven miles N.N.E. of the City Hall, and about a quarter of a mile from the banks of the Hudson River, which it overlooks. It is on one of the most elevated hills, known in history as the "Harlem heights," and commands a prospect which, for extent, variety, and beauty, is rarely equalled.

"The farm contains about fifty-five acres, and is bounded, on its western side, by the Bloomingdale road. About thirty acres of it are under high cultivation, portions being devoted to grass, vegetables, and ornamental shrubbery.

"The part last-mentioned includes a liberal space, which is laid out and planted in one of the most approved styles of English gardening. This having been done in the earliest years of the institution, the trees, of which there is a great variety, have many of them attained their full growth; and as from year to year deficiencies have been supplied and the variety increased, the grounds will favourably compare with most in the country. In short, there are but few, upon this side of the Atlantic, which bear so strong a resemblance to the beautiful homesteads of the wealthy in the rural, cultivated districts of England."

In the moral regimen at this institution, every practicable effort is made to pursue that system, at once gentle, philosophical, and practical, which has resulted from the active and strenuous endeavours of many philanthropists, in the course of the last half century, to meliorate the condition of the insane. The primary object is to treat the patients, so far as their condition will possibly admit, as if they were still in the enjoyment of the healthy exercise of their mental faculties. An important desideratum for the attainment of this object is to make their condition, as boarders, as comfortable as possible, that they may be the less sensible of the deprivations to which they are subjected by a removal from home. Nor is it less essential to extend to them the privilege, or the right, of as much liberty, and as much freedom from personal restraint as is compatible with their safety, the safety of others, and the judicious administration of other branches of curative treatment. The courtesies of civilized and social life are not to be forgotten, tending, as they do, to the promotion of the first great object already mentioned, and operating, to no inconsiderable extent, as a means of effecting restoration to mental health.

On the subject of restraint, the managers of the institution observe—

"They have never, however, become proselytes to the doctrine of the absolutely entire disuse of all restraining apparatus. There are exceptions to all rules which are not governed by the invariable laws of mathematics or of moral right, and no argument, however subtle or specious, or, to appearances, however strongly based, theoretically, upon benevolence, philanthropy, kindness, and the golden rule of 'doing to others as we would, under similar circumstances, that they should do unto us,' can overthrow our belief, founded upon the observation of several years, that there are cases in which the welfare of the patient and the dictates of true humanity require a resort to some restraining means. The truth of this proposition may be, and perhaps is, acknowledged by all. Yet those who, in their recession from left hand defections, have, in our judgment, fallen into right hand errors, assert that, in the cases alluded to, whatever restraint is applied should be that of the hands of the attendants. To this substitute, or subterfuge, we cannot resort, knowing, as we do, the greater irritation produced in a patient by being held by the hands of attendants, than by having his limbs confined by mechanical appliances. In the former, mind struggles with mind; in the latter, with matter alone. The only means of restraint now used in the asylum are the camisole, or long sleeves, leathern muffs for the hands, and the invaluable apparatus, invented by Dr. Rufus Wyman, for confining a patient in bed.

"The camisole is in nearly all cases sufficient. During the last three years, the muffs have not been used in more than two or three cases annually, and in those for but a day or two, or at the most a few days each. There was one period of thirteen months, during which restraint was resorted to but in two cases in the men's department. In one of these, the patient, while in a condition of typhoid delirium, wore a camisole three days, and in the other, the patient's hands were similarly confined a few hours to ensure the vesication of a blister.

"We have found that the proportion of women requiring restraint is greater than that of men, and in this, it is believed, that our experience coincides with that of physicians of other institutions.

"In no less than three cases, in which there was prolonged and exhausing excitement, we are convinced that Wyman's bed apparatus has been the means of insuring sleep, and of saving the life of the patient. It is a method of restraint with which every institution should be supplied."

Previously to 1844, 594 patients afflicted with *delirium tremens*, and addicted to the habitual and excessive use of intoxicating liquors, were admitted into the asylum. Of this number, 511 were males, and 83 females. In the subjoined table, these cases are arranged according to their several admissions and re-admissions.

| | Males. | Females. | Total. |
|---|---|---|---|
| First admission | 274 | 48 | 322 |
| Second admission | 85 | 17 | 102 |
| Third admission | 42 | 7 | 49 |
| Fourth admission | 29 | 4 | 33 |
| Fifth admission | 20 | 3 | 23 |
| Sixth admission | 14 | 2 | 16 |
| Seventh admission | 8 | 2 | 10 |
| Eighth admission | 5 | 0 | 5 |
| Ninth admission | 5 | 0 | 5 |
| Tenth admission | 5 | 0 | 5 |
| Eleventh admission | 5 | 0 | 5 |
| Twelfth admission | 3 | 0 | 3 |
| Thirteenth admission | 2 | 0 | 2 |
| Fourteenth admission | 2 | 0 | 2 |
| From 15th to 26th, each one admission | 12 | 0 | 12 |
| Total | 511 | 83 | 594 |

Fifty-five per cent. of the whole of these cases were born in the State of New York; and 40, or 14 per cent., were natives of other States of the Union. The foreigners admitted, amounted to 81.

"Merchants, traders, clerks, professional men, persons of leisure and young men without employment, furnish one hundred and twenty-nine, or ten more than one half of the two hundred and thirty-eight patients admitted. Without explanation, the necessary inference must be far from flattering to these classes. It should be recollected, however, that they constitute no unimportant proportion of the population of the commercial and wealthy city of New York. They are, moreover, those classes the members of which, more generally than those of other portions of the population, resort to this institution when thus diseased. The great majority of persons whose pecuniary resources are limited, are taken to places where the expenses are less.

*Table showing the Age of 254 Patients.*

| Sex. | Under 20 years. | From 20 to 30 | From 30 to 40 | From 40 to 50 | From 50 to 60 | From 60 to 70 | Total. |
|---|---|---|---|---|---|---|---|
| Males | 3 | 77 | 81 | 36 | 10 | 5 | 212 |
| Females | 0 | 8 | 19 | 7 | 6 | 2 | 42 |
| Total | 3 | 85 | 100 | 43 | 16 | 7 | 254 |

The decennium in which there was the greatest number, is that from thirty to forty years. The next is that from twenty to thirty; and the third, that from forty to fifty.

In the community at large, there is a much greater number of persons between twenty and thirty years of age, than in any decennium of more advanced life. Hence the proportionate number of these patients between thirty and forty years, as compared with the living population of the corresponding age, is much greater than that in any other period of existence.

*Table showing the Civil Condition of* 286 *Patients.*

| | Males. | Females. | Total. |
|---|---|---|---|
| Single | 122 | 1 | 123 |
| Married | 115 | 37 | 152 |
| Widowed | 4 | 7 | 11 |
| Total | 241 | 45 | 286 |

The number of unmarried men exceeds that of the married by seven, although, if the widowed be included with the latter division, the single predominate over the married, by but *three.* This approximation to equality of numbers in the classes of celibacy and matrimony, though so strikingly remarkable with the men, does not obtain with the women. Of the latter sex, the married exceed the unmarried in the proportion of thirty-seven to one, or, if the widowed be included with the married, in the proportion of forty-four to one.

*Table exhibiting the Results of all the Cases of First Admission.*

| Result. | Males. | Females. | Total. |
|---|---|---|---|
| Cured | 244 | 42 | 286 |
| Much improved | 1 | 0 | 1 |
| Improved | 0 | 3 | 3 |
| Relieved | 1 | 0 | 1 |
| Unimproved | 5 | 2 | 7 |
| Eloped | 2 | 0 | 2 |
| Died | 19 | 1 | 20 |
| Remain | 2 | 0 | 2 |
| Total | 274 | 48 | 322 |

As a general rule, delirium tremens soon terminates either in recovery or death.

Considering the severity of the disease, it is eminently curable. Thus, of 322 patients, 286 were cured, and but 20 died. The two still remaining in the house are also cured.

It is proper to remark, as has already been intimated, that in some of these cases, the patient had no delirium while at the Asylum. Among them were a few in each of the various conditions and phases, both temporary and more prolonged, of intoxication.

The rapidity with which the delirium approaches its crisis, in fatal cases, is well illustrated by the following table, indicating the term of residence at the Asylum, of each patient who died.

| Time in the Asylum. | Males. | Females. | Total. |
|---|---|---|---|
| One day | 2 | 0 | 2 |
| Two days | 2 | 1 | 3 |
| Three days | 2 | 0 | 2 |
| Four days | 5 | 0 | 5 |
| Five days | 1 | 0 | 1 |
| Seven days | 1 | 0 | 1 |
| Nine days | 1 | 0 | 1 |
| Ten days | 1 | 0 | 1 |
| Twelve days | 1 | 0 | 1 |
| Thirteen days | 1 | 0 | 1 |
| Twenty-seven days | 1 | 0 | 1 |
| One month and 22 days | 1 | 0 | 1 |
| Total | 19 | 1 | 20 |

"One man was received twelve times more, and discharged *cured* seven times, *relieved* five times. His last discharge was about twelve years since, and during the interval between that time and the present, his habits have been strictly temperate, and his mental condition perfectly healthy."

The following extract on the subject of *delirium tremens* will be interesting to the British psychologist:—

"Of all the diseases to which the human race is subject, there is none that more completely unmans its unfortunate victim, more entirely divests him of all the attributes the possession of which has justified him in assuming the title of "the lord of the creation," than delirium tremens when in the plenitude of its activity.

"The quivering tongue, the disordered stomach, the torpid liver, the rapid pulse, the contracted pupil, the inability to sleep, the irregularity of nervous power, the impotent functions of the brain and the consequent insubordination of the system to its control—these physical symptoms, though much, are but little when compared with the mental phenomena resulting from them. The depraved action of the avenues to the mind,—the external senses—and the unhealthy functions of the perceptive faculties, whence the patient is unable to appreciate or understand the nature or the relations of the objects by which he is surrounded, the entire confusion of his ideas of matter, time, and space, the laws by which they are regulated and the inevitable results of those laws, if not the least alarming, are certainly less prominent and imposing than some of the other symptoms. These may be called the negative mental phenomena. The positive are more salient, and hence make a stronger impression on the beholder. They are the visions which are continually conjured up by a wayward, excited, and ungovernable imagination, more varied in their forms and characters than are the designs of the boldest artist, more diverse and unstable than the ever-changing pictures of a phantasmagoria.

"The walls of his apartment, mere mortar and whitewash to the view of other people, present to the patient pictures of every possible variety in character and composition. Animals of various kinds throng into his room, crouch before him with threatening gestures, and grimaces the most frightful, creep beneath his bed, or crawl upon it with torturing menaces. Enemies in human form spring up to bind, to drag to prison, to the tribunal of justice, to the rack or to the place of execution, or, perchance, to shoot or slay with the sword; and, finally, the phantoms of the ideal world, spectres with gorgon heads, and bodies more hideous than those of the satyr or the fabled tenants of the lower regions, glower upon him with their eyes of fire, gnash their teeth in fiendish defiance, at length seize upon him, and he struggles with them in the full faith that he has encountered the devil incarnate.

"Such are the features which constitute the most distinctive, and, to some, the most appalling characteristics of this disease. How beautiful the results of the harmonious movements of that system which, as the crowning work of the creation, was both "fearfully and wonderfully made," yet how revolting the effects of its discordant action!

"But, as has already been observed and demonstrated, notwithstanding the remarkable physical disorder, and the heterogeneous medley of mental phenomena, attendant upon the malady in question, there are but few acute diseases involving any important organ, or series of organs, which are more curable. The physical powers, though so nearly prostrate, rise with a resiliency which is truly remarkable, and the mind rapidly resumes its healthy action.

"It has already been observed that delirium tremens is not usually considered as ranking under the general head of insanity proper. What opinion soever may be entertained upon this subject, the malady is so different from ordinary mental alienation, in both its characteristics and its duration, that the therapeutic principles adapted to the treatment of the former are entirely inapplicable to the latter. Hence, as well as for reasons hereafter to be mentioned, we have ever held the opinion—and it has been very strongly confirmed by the practical observation of several years—that cases of delirium tremens ought not to be admitted into institutions intended for the insane.

"The disease is of short duration, and consequently the patient requires *absolute* seclusion or close confinement, for but a limited period. The converse of this proposition, as a general rule, obtains with the insane. The internal police of an Asylum cannot, therefore, be well adapted to the necessities of the two classes. The supervision, the restraint, the abridgment of liberty necessary for the one, are not so for the other. If the delirium patients, after recovery from the immediate effects of the disease, be allowed to have all the privileges to which they are entitled, compatible with their condition, ill feeling and jealousy are engendered among the insane, to whom those privileges cannot safely be extended.

"Of the many cases of delirium tremens which have been admitted into this institution, there have been but comparatively few instances of entire reform from the habit of intoxication. Such reformation could not be expected from the brief term of seclusion to which the patients are subjected. Accustomed as the persons have been, in most cases, for many years to the use of liquor, the whole frame, and particularly the nervous system—those organs so mysterious in their organization, so wonderful in their functions, and so difficult of control—have adapted themselves to the stimulus. Every system of organs, every organ, every fibre, every ultimate corpuscle which assists in making up the fabric of the body, and lends its agency in prosecuting the phenomena of vitality, has, as it were, obtained an abnormal appetite which calls loudly and perseveringly for indulgence,—an appetite which cannot be resisted but by a strong effort of the moral power. Hence, the only hope of reformation, in a great majority of cases, lies in a prolonged seclusion, and a compulsory abstinence from stimuli."

The whole number of patients admitted, from the 16th of June, 1821, to the 31st of December, 1844, was 2937; of whom, 1872 were males, and 1065 females. Deducting from these the 594 cases of delirium tremens already analyzed, there remain 2343 cases, of which 1361 were males, and 982 females.

The subjoined table of admissions and re-admissions is interesting:

| | Males. | Females. | Total. |
|---|---|---|---|
| "First admission . . . . . . | 1090 | ... 751 | ... 1841 |
| Second admission . . . . . | 168 | ... 112 | ... 280 |
| Third admission . . . . . . | 46 | ... 35 | ... 81 |
| Fourth admission . . . . . | 22 | ... 11 | ... 33 |
| Fifth admission . . . . . . | 11 | ... 7 | ... 18 |
| Sixth admission . . . . . . | 2 | ... 6 | ... 8 |
| Seventh admission . . . . . | 2 | ... 5 | ... 7 |
| Eighth admission. . . . . . | 1 | ... 3 | ... 4 |
| Ninth admission . . . . . . | 1 | ... 3 | ... 4 |
| Carried over . . . | 1343 | ... 933 | ... 2276 |

| | Males. | Females. | Total. |
|---|---|---|---|
| Brought forward . . | 1343 | ... 933 | ... 2276 |
| Tenth admission . . . . . . | 1 | ... 3 | ... 4 |
| Eleventh admission . . . . | 0 | ... 3 | ... 3 |
| Twelfth admission . . . . | 0 | ... 3 | ... 3 |
| Thirteenth admission . . . | 0 | ... 3 | ... 3 |
| Fourteenth admission . . . | 0 | ... 3 | ... 3 |
| Fifteenth admission . . . . | 0 | ... 3 | ... 3 |
| Sixteenth admission . . . . | 0 | ... 2 | ... 2 |
| Seventeenth admission . . . | 0 | ... 2 | ... 2 |
| Eighteenth admission . . . | 0 | ... 2 | ... 2 |
| Nineteenth admission . . . | 0 | ... 2 | ... 2 |
| Twentieth admission . . . | 0 | ... 2 | ... 2 |
| Twenty-first admission . . . | 0 | ... 2 | ... 2 |
| Twenty-second admission . . | 0 | ... 1 | ... 1 |
| Total . . . . . . | 1344 | 964 | 2308 |

"Thus, although the whole number of CASES was 2308, it appears, by the first admissions, that the actual number of PERSONS was but 1841, of whom there were 1090 males, and 751 females."

It is a point of some interest to ascertain the particular months in which the greatest numbers were brought to the asylum. In order to arrive, as nearly as possible, to an accurate result on this point, it is necessary to reject the admissions in 1821, inasmuch as the institution was not opened until the middle of June. This done, there remains a period of twenty-three complete years. To avoid another cause of error, deduction must also be made of the patients who were brought, *en masse*, from the alms-house, the numbers and time of admission of which have already been detailed in Part First. It is obvious that patients brought in this manner do not fairly represent the ordinary current of admissions.

In several instances, the cases coming from the alms-house were re-admissions. The whole number of first admissions was as follows:—

| | | | |
|---|---|---|---|
| In January . . | 1824, | there were | 8 |
| In August . . | 1831, | „ | 17 |
| In September . | 1831, | „ | 7 |
| In July . . . | 1833, | „ | 24 |
| In January . . | 1835, | „ | 10 |
| Making the whole number . . | | | 66 |

These being subtracted, the monthly admissions, for the twenty-three years, were for—

| | | | |
|---|---|---|---|
| January . . . | 77 | July . . . . | 157 |
| February . . | 90 | August . . . | 151 |
| March . . . | 126 | September . . | 138 |
| April . . . . | 144 | October . . . | 148 |
| May . . . . | 186 | November . . | 134 |
| June . . . . | 202 | December . . | 120 |

The minimum number is in January, and thencefoward there is a regular increase until June, in which is the maximum; and thereafter a progressive diminution, with the single exception of October, throughout the remaining months.

Various authors who have written upon mental diseases, as well as other physicians who have devoted much time to the treatment of them, have endeavoured to ascertain the extent of the influence exerted by the different seasons of the year, as a generative or exciting cause of insanity. It is reasonable to suppose that, other things being equal, the change of temperature from winter to spring, and the heat of the warmer months of the year, acting, as they do, as causes of debility in the human frame, and, the latter particularly, inducing a greater indulgence in beverages of a deleterious nature, as well as congestion of the liver and some other physical disorders, should operate as productive causes of mental alienation.

For the purpose of accurately illustrating this subject by the cases before us, they are here arranged according to the several seasons:

| Spring. | Summer. | Autumn. | Winter. |
|---|---|---|---|
| March . . 126 | June . . . 202 | September, 138 | December, 120 |
| April. . . 144 | July . . . 157 | October. . 148 | January . 77 |
| May . . . 186 | August. . 152 | November, 134 | February. 90 |
| Total, 456 | Total, 510 | Total, 420 | Total, 287 |

It will be perceived that the greatest number was in summer, the next in spring, the third in autumn, and the least in winter.

By assembling in one class, the six months from April to September inclusive, that half of the year in which the temperature exercises its greatest influence, and in another class, the six months from September to March, when that influence is least, the contrast is rendered still more apparent. The number in the former period is 978, and in the latter, 695. This shows an excess of 40 per cent. in the season of the highest temperature.

Since, in a large majority of cases, some time elapses after the first invasion of insanity before the patient is removed to the Asylum, it is obvious that the month, or season of admission, does not always correspond with that of the commencement of the disease, which is the period desirable to be obtained. Again, the more favourable weather, and the greater facilities for travelling, during the warmer months, are inducements to people residing at a great distance to select that season for bringing patients. In regard to this institution, however, no inconsiderable portion of the cases were of but short duration, and a very large majority of them lived in such near proximity to the asylum as to render access to it at all times easy. This table, therefore, assumed as a criterion of the general prevalence or occurrence of insanity, would not be so inaccurate as under other circumstances it might have been. It undoubtedly is somewhat modified by the influences mentioned.

"The number of males exceeded that of females in the ratio of 1090 to 751, or of 145 to 100."

"The age of but 1710 patients was ascertained. These are arranged in the following table:—

| | Males. | Females. | Total. |
|---|---|---|---|
| Under twenty years | 65 | 51 | 116 |
| From twenty to thirty years | 359 | 268 | 627 |
| From thirty to forty years | 292 | 171 | 463 |
| From forty to fifty years | 157 | 113 | 270 |
| From fifty to sixty years | 87 | 66 | 153 |
| From sixty to seventy years | 37 | 19 | 56 |
| From seventy to eighty years | 17 | 5 | 22 |
| From eighty to ninety years | 1 | 2 | 3 |
| Total | 1015 | 695 | 1710 |

"The number in the decennium from 20 to 30 years is much larger than that in any other similar period, and exceeds that of the next highest decennium, which is that from 30 to 40 years, by 35 per cent. The several periods—whether reference be had to the total numbers, or to those of either sex separately—if arranged according to the numbers in each, beginning with the highest and regularly descending, are as follows:—

1st. From 20 to 30 years.
2nd. From 30 to 40 years.
3rd. From 40 to 50 years.
4th. From 50 to 60 years.
5th. Under 20 years.
6th. From 60 to 70 years.
7th. From 70 to 80 years.
8th. From 80 to 90 years.

"Omitting the cases under twenty years, there is a regularly progressive diminution throughout the several decades included in the table.

"Assuming, therefore, these data as the exponent of the whole insane population of the country, it is evident that mental diseases are far more prevalent in the period from 20 to thirty years of age, than in any other epoch of equal duration in human life."

It is important and interesting to investigate the civil condition of the insane. On this subject the volume before us contains some interesting information.

"The influence which position in life, in regard to celibacy and marriage, exerts as a cause of mental disease, became, long since, a subject of inquiry. So far as this position was ascertained, relative to the patients at this institution, the results are as follows:—

| | Males. | Females. | Total. |
|---|---|---|---|
| Single . . . . | 570 | 273 | 843 |
| Married . . . . | 432 | 358 | 790 |
| Widowed . . . | 36 | 80 | 116 |
| Total . . | 1038 | 711 | 1749 |

"Of 1749 patients, 843 had never been married; 790 were married, and their partners living; and 116 were widowed. Thus, the single exceed the married, exclusive of the widowed; but if the latter be included, the married exceed the single.

"In men, the number of the single is greater than that of both the married and widowed by 102, which is equivalent to 18 per cent. On the contrary, with the women, the married whose husbands were living, far exceeded the single, and including the widows, that excess is 165, or a little over 60 per cent.

"This difference in regard to the sexes is, to us, both remarkable and unexpected. The fact that among the insane admitted into this establishment, the number of unmarried men should predominate so much over the married, while, with the women, the reverse, to a very remarkable extent, should obtain, can only be accounted for in the supposition that, in the community whence these persons mostly came, there are peculiar influences operating upon the public health.

"In regard to females, there are special causes which may, at all times and in all countries, render the number of married greater than of single among the insane; although we are not aware that this has ever been demonstrated to be true, yet it is to be feared that for the origin of so great a discrepancy of numbers, research must be made into the habits and customs, the physical, mental, and moral condition of the people of New York city. If this be true, it is not believed that those causes are peculiar to New York alone, but that they are common to all great commercial settlements, and thus furnish melancholy evidence, to the full extent of the signification of the assertion, that—

'God made the country, but man made the town.'

"Of 1038 men, but 36 were widowers. This is equal to about 3½ per cent. Of 711 women, 80, or a little more than 11 per cent. were widows. Hence, the relative proportion of the latter was more than three times greater than that of the former. It is, we believe, a well ascertained fact, that, of the widowed in the general population of the northern states, there are more women than men; yet that predominance cannot at all account for this disproportionate number among the insane. Grief, of which the female mind is more susceptible than that of man, and the comparatively unprotected and dependent condition in which widows are placed by the loss of their partners—a position peculiarly exposing them to other exciting causes of mental disorder—must, it is believed, be the principal sources from which have arisen this large number of insane among widowed females."

With regard to the occupation in life of the 1015, whose cases are recorded, the following stand at the head of the list:—

| | | | |
|---|---|---|---|
| Merchants and traders | 111 | Clergymen | 10 |
| Clerks | 62 | Sea captains | 26 |
| Physicians | 20 | Farmers | 193 |
| Lawyers | 16 | Sailors | 19 |

"Of 1015 patients, 204 were merchants, traders, and their clerks. This is little more than 20 per cent. of the whole.

"Of professional men and others engaged chiefly in mental occupations, there were 106, or a trifle more than 10 per cent.

"From the army and navy there were 11, which is a small fraction more than 1 per cent.

"Of mariners, the number was 52, or a little more than 5 per cent.

"In the class of persons engaged in active employment out of doors, there were 322, or 31 and 7·10 per cent. This class, however, includes people of various occupations, in both city and country; 212 of them were directly engaged in agricultural pursuits. This is a small fraction more than 20 per cent. of the whole number. The remainder were mostly from the city, although, under the term 'labourer,' it is probable that some from the rural districts are included.

"In the class of active employment within doors, the number is 111, or something more than 10 per cent.

"This division also includes a great variety of occupations. Of carpenters, cabinet-makers, and other workers in wood, there were 64, or 6 and 3·10 per cent. of the whole number. Much of the labour of carpenters is out of doors, but it was thought they were more properly arranged under this class than any other.

"In the class of persons whose employment subjects them, in a greater or less degree, to an elevated artificial temperature, there were 37, or 3 and 6·10 per cent.

"Of those who, in their vocation, are subjected to the deleterious action of metals, either in the form of vapour or otherwise, there were 16. This is about 1 and 6·10 per cent.

"There were 32 dealers in liquor, a number equivalent to 3 and 1·10 per cent.

"It may be remarked that, under the term 'grocer,' are included some whose principal business was the mixing and retailing of spirituous liquors.

"In that division, the occupations of which are mostly of a sedentary character, there are 68, or 6 and 7·10 per cent.

"Of men of leisure, and young men without employment, there were 55, or 5 and 4·10 per cent.

OCCUPATION OF FEMALES.

"Of the 751 female patients, the condition, in regard to occupation, of but 241 is recorded. Of these, the most important are as follow:—

| | | | |
|---|---|---|---|
| Sempstresses . . . . | 52 | Workers in factory . . | 2 |
| Servants . . . . . | 51 | Hucksters . . . . . | 2 |
| Teachers . . . . . | 9 | Pawnbroker . . . . | 1 |
| Grocers and tavern-keepers . . . . . | 5 | Cook . . . . . . . | 1 |
| | | Farmers' wives . . . | 24 |
| Nurses . . . . . . | 3 | Mechanics' do. . . . | 11 |
| Paper-folders . . . . | 2 | Farmers' daughters . . | 20 |

"The rest were mostly the wives and daughters of persons engaged in a variety of employments. Of the 510 whose occupation is not stated, it is probable that most of them were from the non-labouring classes of society."

We now come to the consideration of the CAUSES OF INSANITY.

The subject of hereditary predisposition is first spoken of. It appears that of eighteen hundred and forty-one patients, three hundred and twenty-three—of whom one hundred and eighty-seven were males, and one hundred and thirty-six females—are recorded as having one relative or more insane; this is equivalent to seventeen and a half per cent. This per centage in each sex, taken separately, is as follows: men, 17 and 16·000; women, 18 and 11·000.

It is not to be presumed, however, that this is even a near approximation to the number actually having relatives of disordered mental powers. During the first few years of the existence of the Asylum, there appears to have been but little attention paid to this particular subject, and hence the records thereupon are imperfect. There are other important obstacles in the way to a correct knowledge of the full extent to which the hereditary predisposition prevails among the patients admitted into a public institution. These obstacles may, by perseverance, be measurably overcome.

Insanity being a disordered manifestation of the mind, dependent upon some disease of the body, either functional or organic, is governed by the same laws as many or most other maladies to which the human race is subject. Like consumption, gout, diseases of the liver and the heart, it may attack any person whatever, but it is certainly somewhat more likely to prevail among those whose ancestors have suffered from it.

Of the men included in the foregoing table, one hundred and eighteen inherited the predisposition from direct ancestors, and thirty-three of these had other relatives insane. Of the remainder, 68 had collateral relatives insane, but no direct ancestors; and one had a child insane. Of the 52 who had insane parents, it was the father in 27 cases, and the mother in 25. In one of these, both father and mother had been deranged. It is also stated, that two of those included under the term hereditary had ancestors, both paternal and maternal, who were subject to the malady.

Of the women, the predisposition was transmitted from direct ancestors in 89; of whom 67 had other relatives insane. In the remaining 42, the disease is stated to have appeared only in persons collaterally connected; and in five cases in their children alone. There are 18 cases in which it is mentioned that the father was insane. In one case, the father and mother were both deranged. In the case where it is asserted that the whole family were insane, it is said that all her father's family, which consisted of 12 children, have been deranged, and that their insanity did not, in a single instance, make its appearance before the age of 21 years. Two of her brothers, while insane, committed suicide. None of the third generation have yet been attacked with mental disorder, although several of them have passed the age at which it made its appearance in the second.

Of the patients whose disease was supposed to have originated from physical causes, there were 664; of whom 379 were males, and 285 females. Of those supposed to have arisen from moral causes, there were 522; 310 males, and 212 females.

Almost all the older authors upon insanity believed that mental causes were more prolific of the disease than physical. Within a few years, however, the opposite opinion has been gaining ground—an opinion which is sustained by these statistics.

It will be perceived that, although a distinct class has been made of all the cases of delirium tremens, intemperance occupies the highest rank, in point of numbers, among the physical causes. So far as this item is concerned, the table may undoubtedly be taken as a criterion by which to judge of the comparative influence of the various agents productive of insanity in the United States of America.

Thirteen cases, of which five were men and eight women, resulted from the excessive use of opium. In one of the men, the cause was more fully stated as the "too abundant indulgence in opium, snuff, and tobacco." The action of these narcotic substances upon the nervous system is very similar to that of alcoholic liquors; and a recent French writer not only maintains that this action is precisely the same, but asserts that he has proved it to be so. If, therefore, one of the necessary effects of alcohol is to establish in the system a condition which will prevent the healthy action of the mind—and we are but too well aware that this is the fact—it follows that the narcotics in question would produce an identical effect, and cause insanity. No one, it is presumed, will question the truth of this proposition, so far as relates to opium. In reference to tobacco, there may be some doubt. Several modern authors, however, concur in the belief that, when excessively used, it may be the principal cause of mental derangement, and cases thus produced have been reported at a number of institutions.

The immediate action of this substance upon the nervous system, in persons of a highly excitable temperament, is so powerful that, when smoking, they feel a peculiar sensation, or thrill, even to the remotest extremities of the limbs. A constant stimulus of this kind, upon a nervous temperament, can hardly be otherwise than deleterious. Tobacco, particularly when used by smoking, tends to disturb the functions of the liver; and disordered action of this organ is not an unfrequent cause of mental disease. It also produces, or assists in producing, a chronic inflammation of the mucous membrane of the alimentary canal. The inflammation of this membrane may become the cause of mental disturbance. Again, particularly in persons in whom it excites an inordinate secretion from the salivary glands, tobacco is likely to produce dyspepsia, a disease which, more than almost any other whose action is sympathetical upon the brain, affects the manifestations of the mind.

Who has not experienced or observed this deleterious influence, producing depression of spirits, dejection, taciturnity, and inability to contend with the cares of life; gloom, despondency, and perhaps a disposition to self-destruction, or actual insanity in the form of melancholia?

How little or how much soever tobacco may act, either immediately or remotely, as a generative cause of insanity, it is a fact well known to all connected with public institutions of this kind, that there is no stimulant or narcotic substance in which the insane are more prone to indulge. If within their reach, those who, previously to becoming insane, have been accustomed to it, will use it to excess, and many or most of those who have not before been addicted to the habit, soon fall into it. One man, included among the patients remaining in the institution at the time these statistics close, kept constantly in his mouth, both day and night, excepting when at meals, a quid of tobacco, frequently nearly as large as an ordinary hen's egg. Whatever saliva it might have produced, was rarely, if ever, ejected from the mouth, but usually swallowed. He had been in the institution during the whole period of its existence, being one of those who were brought from the old asylum. He had been accustomed to the habit for many years; and it might almost be said of him—

> " Like to the Pontic monarch of old days,
> He fed on poison, and it had no power,
> But was a kind of nutriment."

Although so completely insane and incoherent as it is possible for a human being to be, he worked regularly, doing about as much as any ordinary labourer. The tobacco appeared to have a soothing and controlling effect upon him, enabling him to concentrate his powers upon the labour in which he was employed. If deprived of it for a few hours, he became restless, agitated, excited, talkative, and unable to apply himself to his occupation. In this respect, the narcotic had an effect upon him, opposite to that which it produces upon many of the insane. It frequently increases their excitement, and, in some instances, to a remarkable degree. Its action, upon the whole, is considered so deleterious, that, in most of the well-conducted establishments for the insane in this country, its use among the patients is prohibited. At this institution it is not permitted, excepting in a few cases, in small quantities, by patients who have resided there many years.

There are sixty-nine cases included under the several causes, the names of which imply an organic lesion of the brain or its membranes. According to our belief, there is always cerebral disease in insanity; and such alone has the power to affect the manifestations of the mind. In some cases this disease is organic, but in the majority merely functional, the healthy action of the brain being disturbed by its intimate sympathy with other organs which are diseased. In many cases, it is absolutely impossible for the most experienced and expert observer to decide, in the early stages of insanity, whether the disorder of the brain be organic or functional. Hence, it is possible that the number of cases here attributed to the several diseases of the brain, is not sufficiently large.

Thirty-one cases are recorded as having originated from injuries produced by falls. The effect of sudden shocks or concussions of this kind, falls most heavily upon the brain and nervous system. Hence their agency in the production of mental disorder is most obvious.

If the prick of a pin or needle exert, as it frequently does, so potent an influence upon the nervous system as to result in that terrible disorder popularly known as the lock-jaw, it is certainly not surprising that a punctured or gun-shot wound should cause insanity. One case, arising from each of these causes, is mentioned in the Report before us.

One case is also recorded as the effect of a kick by a horse, upon the region of the stomach. Here the disorder of the brain was undoubtedly secondary to the immediate effect upon the great central plexus of the sympathetic nerve, in the region receiving the shock.

After the cases of isolation, there is a series of causes, all, or nearly all, of which exhaust the nervous power, occasion debility, and probably by this means destroy the healthy exercise of the brain. The first of these is masturbation. Thirty-seven cases are placed against this as their exciting cause. For a long time this has been known as one of the many agents tending to destroy the balance of the mind, but it is not until within a few years that its influence was supposed to be so great as it is at present by most physicians to institutions for the insane. Although it is acknowledged to be a prolific cause, yet there is danger of misapprehension upon this point. The habit is, undoubtedly, in many cases, the *effect* of the disease.

The important revolution which the system of both males and females undergoes at the time of puberty, sometimes seriously affects the mind and produces absolute insanity. The tendency of this change to operate upon the healthy action of the mental powers is greatly increased by the simultaneous disposition to rapidity of growth. When the nutritive vessels are acting with such energy, and all parts of the frame are becoming developed with an unwonted rapidity, the texture of the body is loose, incompact, and light, wanting the density, tone, and stability essential to a vigorous performance of its functions, and the nervous fluid cannot act with the celerity and vigour requisite to perfect health.

Four cases of men and seven of women are attributed to excessive bodily exertion and loss of sleep.

The renovation of energy by sleep is absolutely essential to the healthy exercise of both the physical and the mental powers. So important is its position as a preventive to mental derangement, that were we called upon to give advice to all who are predisposed to insanity, are threatened with it, or fearful of it, and were we obliged to give that advice in the briefest possible terms, we would concentrate it into an imperative phrase of but two words, "sleep enough."

Nothing exhausts the nervous energies of the system more rapidly than constant and prolonged watching. It subverts a primary law of nature—a law which cannot be seriously infringed with impunity.

Excessive bodily exertion wearies the frame by its heavy tax upon the nervous system. The muscles, it is true, are the immediate organs of motion, and consequently of labour, but they are inert and incapable of movement if deprived of the nervous stimulus. If a constant supply of the latter could be continued for an indefinite period, we can perceive no sufficient reason why the muscles should not perform their office with all their energy unweariedly. At least, the converse of this proposition has never, so far as we are informed, been demonstrated.

Inordinate and prolonged labour reduces the nervous energy, and rest and sleep become necessary to its renewal. But it is frequently reduced to so low a point that sleep becomes impossible, or if at length it be attained, it is imperfect, broken, and insufficient to enable the nervous system to rally its wonted forces. Hence, in these cases, it may be not so much the bodily exertion itself as its secondary effect, the deprivation of sleep, which is the immediate cause of mental disorder.

One case is said to have arisen from "Mesmerism." This was the cause assigned by one of the parents of the patient. The leading features in the history of the case are as follows:—The patient was a young man, about twenty years of age, of a highly nervous temperament, with a brain remarkably developed and corresponding intellectual powers. For several years he had suffered from occasional epileptic fits, which as yet had left his mind but little if at all impaired. The skill of many physicians and the virtues of every medical resource believed to be applicable to such cases had been exhausted upon him without benefit. As a dernier resort, and at a period when he was in a state of comparative stupor, such as frequently follows a succession of epileptic fits, he was placed under the care of a person professedly practising "Mesmerism" for the cure of disease. To use the expression of this person, "The patient was magnetized daily for nearly a month" without effect, he remaining in the torpid condition already mentioned. At length, he was suddenly roused, appeared rational for a few hours, and then passed into a state of high excitement and absolute mania. A day or two afterwards he was brought to the asylum, with his arms and legs strongly bound. When admitted he talked but little, and that little was perfectly devoid of meaning. He was highly excited; his face flushed, and the veins of his head swollen; the circulation rapid, the pulse being from one hundred and twenty to one hundred and forty per minute; the tongue furred, and the bowels very much constipated. After free catharsis—an inordinate quantity of medicine being required to operate upon his bowels—he was placed upon the use of sedatives. Under this treatment, and after the lapse of two days, he began to improve, and in eight days he left the asylum, restored to his ordinary condition, and without much of the torpor that existed previously to his excitement.

The general term *ill health*, under which thirty-seven cases are arranged, is so vague and indefinite, and it may include so great a variety of diseases, that it is susceptible of but little comment of special application. In general terms, it may be supposed that almost any malady, if sufficiently prolonged, may impair the vigour of the body, act sympathetically on particular organs, diminish the quantity or derange the action of the nervous fluid, and thus disorder the manifestation of the intellect.

The next series of cases are those which are arranged under the generic term *fever*. Those are placed first whose predominant pathological effects are upon the circulatory and nervous systems; and those which follow have, as a leading feature, disordered action of the liver.

Pure fever, unallied with a pathological condition of either the nerves or the liver—if, indeed, such a disease exist—may, from the rapidity and force of the circulation, impair the functions of the brain, or it may produce the same result sympathetically, through the inflammation of the mucous membrane of the alimentary canal.

If the disease be of the typhus or typhoid form, in which the nervous system becomes most seriously involved, and delirium is frequently an accompanying symptom, it is easily comprehended that mental disorder of a more permanent character may ensue.

It is probable that of the thirty-one cases included under the general term fever, the disease in many or most of them was of one of the specific forms afterwards mentioned.

In the bilious fevers, it appears to us that the disordered action of the liver is the primary cause of insanity when this disease ensues.

Whether the disordered action of the brain in these cases, arise from sympathy with the liver, or be produced by the condition of the blood—modified as that fluid is, in its constitution, so far as regards the elements of the bile—is a question which we pretend neither to explain nor to understand.

Twenty-six cases are said to have arisen from dyspepsia. The remarks already made upon this disease preclude the necessity of any further comment.

Rheumatism and gout, undoubtedly, as a general rule, cause insanity by a metastasis to the dura mater, the fibrous membrane covering the brain.

Phthisis pulmonalis, or the true consumption, is not unfrequently connected with insanity, either as a cause, a concomitant, or possibly, in some instances, an effect. In the whole range of human maladies, there are but few cases more singular or interesting than those in which these two diseases alternate with each other in the same patient. The consumptive person becoming insane, the progress of the pulmonary complaint is arrested until he recovers from his mental disorder, when it resumes its march until stopped by another attack of mental derangement, again to progress, if that malady be cured, and again to be suspended if the patient should become insane. This singular alteration is probably in obedience to a general physiological or pathological law, that two important and active diseases cannot simultaneously exist and run their natural course.

The deleterious effects of the sudden suppression of a natural secretion, or an accustomed discharge, whether natural or artificial, are well known. Habituated to a constant drain, the body is brought into a condition in which that drain appears necessary for the support of health. If it be suspended, the system becomes plethoric, or laden with matter unqualified to assist in the action of the different organs, and therefore an obstacle to the faithful performance of that action. The brain, in common with other organs, is affected, and consequently the manifestations of the mind disordered.

Some of the eruptive fevers, particularly measles and scarlatina, are proverbial for the physical defects which follow in their train. Their results being thus unfavourable to the perfection of the body, it is not remarkable that they should, in some instances, disorder the action of the intellect. In the foregoing list, thirteen cases are imputed to them.

That mysterious and peculiar influence of the salts of lead which, in some cases, produces *colica pictonum*, a disease so common among painters as to have derived its name from them, is undoubtedly the same which, in other cases, among people who are accustomed to work in those substances, originates insanity.

The case attributed to the inhalation of prussic acid is that of a man engaged in the manufacture of fancy soap. If that acid were truly the producing cause of the disease, it may be supposed to have effected that result by the depression of the nervous power, its natural physiological effect.

The last ten items in the table of physical causes constitute a series of influences to which the female sex alone is liable. We have long held the opinion that in their sex, these are the predominating causes of mental alienation—an opinion corroborated by these statistics. It will be perceived that of two hundred and eighty-five cases of females whose disease is attributed to physical causes, no less than one hundred and fifty-five are arranged in the series in question. The nervous system being more fully developed, at least so far as intensity of action is concerned, in females than in males, and the intimacy between the uterus and the other organs of the body being so intimate, so powerful, and so controlling, as the observation of physicians shows it to be, there is little reason to marvel that the causes in question should be so prolific of mental alienation. Dr. Rush appears to have correctly estimated the potency of these causes, and alleged the fact as an argument in support of the doctrine that women are more subject to insanity than men.

Connected as the Bloomingdale Asylum is with a city almost purely commercial—a city, the majority of whose active adults are subject to the cares, the perplexities, and the fluctuations of trade, it is not remarkable that, among moral causes, pecuniary difficulties should occupy the most prominent position. Under this head there are one hundred and eighteen men and fifteen women, a total of one hundred and thirty-three; and if, as may be most proper, the eleven cases assigned to "the want of employment" be included, the total will be one hundred and forty-four. There is, perhaps, no mental influence which, if examined in all its bearings and relations, exercises so extensive and controlling a power upon man in civilized countries, and more particularly in the United States, as that arising from his pecuniary condition. Connected with this are many if not all his hopes and schemes of ambition, preferment, and aggrandisement—all his prospects of present and future temporal comfort, and all his affections that are enlisted in the welfare of the persons constituting his domestic circle.

A constant business, moderate in extent and sufficiently lucrative to afford a liberal subsistence, can never, in a mind well regulated, operate as an exciting cause of mental disorder. The sources of the evil are, on the one hand, ambitious views and endeavours rapidly to accumulate wealth, and, on the other, the extremes of excessive business, of bankruptcy, and of poverty, the fluctuations and the unwholesome disposition to speculation. Of the one hundred and eighteen cases of men arranged under the head of pecuniary difficulties, the disease in three was attributed to excess of business; in two, to retiring from business; in four, to a sudden access of fortune; in one, to speculation in stocks; and in two, to speculation in *morus multicaulis.*

Moral philosophy requires not, for its illustration, the assistance of the fable of the lion and the gad-fly, when so harmless and apparently impotent a vegetable as the mulberry can overturn the faculties of the human mind.

The moral cause which ranks next in point of numbers, among both the men and women, is the anxiety and other mental influences in reference to religion. The whole number attributed to these is ninety-three, of whom fifty-one were males and forty-two females. Although there were more men than women, yet the proportionate number, when compared with the whole number of admissions, is greatest in the latter.

In a country like the United States of America, of universal toleration upon religious subjects, and sheltering under this broad banner congregations of almost every sect that has ever appeared in Christendom, it is to be supposed that the religious sentiment would act under its greatest possible variety of phases, and in every diversity of gradation between the extremes of apathy and fanaticism. The accurate observer of the events of the last twenty years, to say nothing of a period more remote, cannot fail to have perceived that this is actually the fact. Under these circumstances, and when we consider the whole scope and bearing of this sentiment, both temporal and eternal, we cannot but perceive how important an influence it may exert. It is difficult to believe that "pure religion and undefiled" should overthrow the powers of the mind, to which it was intended to yield the composure of a humble hope and the stability of a confiding faith. Nor do facts authorize any conclusions thus hostile to Christianity, for a great majority of the cases of insanity attributed to religious influence can be traced to the ardour of a zeal untempered with prudence, or a fanaticism as unlike the true religion which it professes, as a grotesque mask is to the face which it conceals. The exciting doctrines of Miller, the self-styled prophet of the immediate destruction of the world, gained but little hold of the public mind in the vicinity of the Bloomingdale Asylum, but in those sections of the country where they obtained the most extensive credence, the institutions for the insane became peopled with large numbers, the faculties of whose mind had been overthrown thereby.

The passions or emotions whose activity tends to depress the energies of both mind and body, may be considered, on strictly physiological principles, as powerful agents in the production of mental disease.

Remorse is the first of these mentioned in the table, and eleven cases, of which five were males and six females, are attributed to it.

Grief, caused by the death of relatives, stands next in position, but first in point of numbers, including, as it does, forty-three cases, of which sixteen were males, and twenty-seven females. Of the men, the particular relatives whose death was followed by so unfortunate an occurrence, is stated to have been the wife in six cases; the wife and child in one; the wife and five children in one; the child in three; the mother in two; the sister in one; and the brother in two.

Of the women, it was the husband in five cases; the child in eight; the father in one; the mother in one; the mother and child in one; the mother and sister in one; the sister in one; the brother in two; and the brother and sister in one.

Forty cases, twelve males and twenty-six females, are recorded as having originated from disappointed affection.

Home-sickness, or, technically, *nostalgia*, is assigned as the cause in three cases—two males and one female. The latter was a Swiss girl who had been but a short time in this country, and could not speak English. Separated from her friends and surrounded by strangers, her spirits were most oppressively borne down by that disease—if disease it may be termed—so proverbial among her countrymen when removed from the sight of their native mountains and valleys, and beyond the hearing of the *rans des vaches*. After a residence of a few days at the asylum, a victim at once to the delusions of insanity, and to the harrowing emotions from which that disease originated, she ended her temporal sufferings by suicide.

Fear is at all times a depressing emotion, whether it be constant and prolonged, or sudden and transient, as more particularly implied by the term *fright*. In the latter case, it is powerfully so, even to the production, in some instances, of immediate death. Its natural effect, and the power of its action, particularly qualify it as a source of mental disturbance, and hence it should at all times, if possible, be avoided. The tales of horror conjured up to amuse or to subjugate children in the nursery, have not unfrequently been attended with the most deleterious consequences; and persons who, for amusement, attempt to frighten or startle their friends, incur the risk of doing the latter an injury beyond their power of reparation.

During the prevalence of an epidemic, the fatality of the disease is greatly augmented by the panic that seizes upon the mass of the community, the depressing influence of which upon the energies, both physical and mental, prepares the way for an easy invasion of the disorder. This influence may also affect the healthy action of the mind. Thus, of the nineteen cases alleged to have been produced by this general cause, two are attributed to the fear of the Asiatic cholera.

With students, whether young or of middle age, if a proper equilibrium be maintained between the physical powers and the intellectual faculties, the development and energies of other portions of the body being so promoted and sustained by exercise, that they may preserve their due relations with an enlarging brain, there need be no fear that mental alienation will result from application to study; but unless this precaution be taken, the midnight oil consumed as a beacon light to guide towards the temple of truth, may become an *ignis fatuus* leading the mind into the labyrinth of insanity. Even in persons of strong constitution and of great physical strength, severe and prolonged study exhausts the nervous energy and impairs the functions of the brain. How much greater must be these effects in a frame naturally delicate, and how much more alarming still, if the body be debilitated by the want of exercise!

In the table of causes, thirty cases are set down as supposed to have been induced by mental application.

Of the two cases placed against the term *mental shock*, one is represented to have been produced by the hearing of good news.

Domestic trouble ranks high among the moral causes. It includes 42 men and 23 women—a total of 65.

Under the general and somewhat indefinite term *anxiety*, there are 22 cases—12 of men, and 10 of women. In two of the men, the anxiety was on account of a false accusation of seduction; and in five others, it was in reference to annoying law-suits in which they were engaged.

Eight cases are attributed to faulty education and parental indulgence. These are subjects which, during the past few years, have been fully discussed by several able writers on insanity, and hence require no extended comments on the present occasion. Although sympathizing deeply in the feelings of the young, and entertaining a pleasing and affectionate emotion for all that cross our path who as yet tread but the vestibule of the temple of life, and ardently wishing to promote, by every judicious measure, their welfare, yet we must, and even for those very reasons, subscribe to the doctrine of the prophet of olden time, "It is good for a man that he bear the yoke in his youth." Let not that yoke, however, be imposed with despotic hands, but with that prudent combination of kindness and firmness which will render its burden light.

Three cases are attributed to undue indulgence in the reading of novels. Inasmuch as this subject has heretofore often received, and undoubtedly will continue to receive the attention of men who "stand in wisdom's sacred stole," we dismiss it without comment.

There are several heads included in the tables, to which especial reference has not been made, but they are either so unimportant, or so similar to others which have been noticed, that they do not appear to call for any specific remarks.

In speaking of the form of disease, the writer observes:

"Mania occupies the first rank in point of numbers, in either sex, as well as in the aggregate. There were nine hundred and five of this form of disease, which is equivalent to *fifty-two and one half per cent.* of the whole number.

"The second in rank among the men, and in the total of the two sexes, is Dementia. Of this, there were two hundred and thirty-five cases, or *thirteen and sixty-five hundredths per cent.* of the whole.

"Monomania occupies the third position in the men, and in the total; but the second in women. There were two hundred and thirty-two cases, or *thirteen and fifty-nine hundredths per cent.*

"Melancholia is the fourth in men, and in the total, but the third in the women. Of this there were one hundred and eighty-five cases, or nearly *ten and three quarters per cent.*

"Epilepsy holds the fifth rank, there being thirty-five cases, or *two and three hundredths per cent.*

"The nosology of mental diseases is still so imperfect, that it is difficult to make an arrangement of cases which would be of any material value, either practical or theoretical. Indeed, there are scarcely two physicians who would classify a series of cases, such as are admitted into any institution, in precisely the same manner. The forms of disease, in the cases included in the foregoing table, were recorded, in part, by several physicians, whose views upon the subject may have differed, and hence the classification is undoubtedly different from what it would have been, had it been made entirely by one. A case called Partial Insanity by one person, might be termed Monomania by another. That which one records as Monomania, another would place under the head of Melancholia. There being no definite line between Mania and Dementia, a given case might be placed under the former by one physician, and under the latter by another.

"A perfect nomenclature of Insanity is a great desideratum."

"With regard to previous attacks of insanity, it would appear that 208 patients were known to have previously suffered from the disorder. This is equivalent to *eleven and twenty-nine hundredths per cent.* of the whole number admitted. The per centage of men is a small fraction more than *ten;* that of women, a little more than *thirteen.*"

"It would appear that 57 men and 52 women, a total of 109, had actually attempted to destroy themselves. The number who had attempted to do it more than once was thirty-eight, of whom 19 were men and 19 women."

Persons who are intimately acquainted with the insane, will recognise an important difference between the terms *disposed to,* on the one hand, and, on the other, *talks of,* or *threatens to* commit suicide. Patients in whom the propensity to self-destruction is the strongest, and who are most likely to be urged onwards by it to a fatal execution of their designs, never, or rarely, in any manner allude to that propensity, but, on the contrary, use every precautionary measure to conceal it. I have never known but one person accustomed to talk of the propensity, who afterwards committed suicide. This was a woman whose case offers an exception not only to this, but to another general rule. The suicidal rarely—so very rarely that, it might almost be said, never—put their intentions into execution in the presence of another person. This patient hung herself within five feet of another female, a fellow-patient, who, however, it is possible she supposed to have been sleeping, as the act was committed in the night.

Patients who *threaten* to commit suicide, almost invariably do so for the purpose of frightening the people around them, rather than from any propensity in that direction. I do not recollect ever to have met with a single exception to this.

One of the most remarkable characteristics of the really suicidal, is their fearfulness of being injured by others. A man will shrink from those by whom he is surrounded, lest they should do him harm, in the slightest degree, and the next moment take his life with his own hand.

The following table will give our readers an idea of the patients who manifested the homicidal propensity:

| | Males. | | Females. | | Total. |
|---|---|---|---|---|---|
| "Had committed murder . . . . | 5 | ... | 0 | ... | 5 |
| Had attempted to kill . . . . . . | 13 | ... | 7 | ... | 20 |
| Threatened to kill . . . . . . . | 11 | ... | 6 | ... | 17 |
| Attacked a daughter while asleep . | 0 | ... | 1 | ... | 1 |
| Thinks it her duty to kill some one | 0 | ... | 1 | ... | 1 |

"Of the five actual homicides, one killed a child; one his mother-in-law; and one, his wife's brother. Of the remaining two, it is merely stated that one killed a person; and the other killed a man with a knife, was tried, and acquitted on the ground of insanity.

"Of the men who attempted to kill, that attempt was made on a wife, by five; on a wife and child, by one; on a father, by two; on a brother, by one; on a sister, by one; and on a constable, by two.

"Of the men who threatened to kill, the threat was against a wife in two cases, his friend in eight, and family in one.

"The attempts to kill, by women, were in five cases upon a child, in one case upon a sister, and in one upon a sister and step-mother.

"The threats to kill, by women, were against a husband in two cases, against her children in one, her friends in one, and in one the particular persons are not mentioned.

"It may be observed that many of the insane, particularly when excited, threaten to kill those around them, without any intention, certainly any *permanent* intention to put their threats into execution."

Relative to the condition of the patients when discharged from the Asylum, it would appear that:—

"One thousand seven hundred and sixty-two patients were discharged—of whom 1046 were men, and 716 women. Of these, 408 men and 264 women were cured, making a total of 672.

"There were 42 of the foregoing patients—23 men and 19 women—who, after a short residence in the Institution, were discharged, as follows:—

| | |
|---|---|
| Much improved . . . . . . . . . . . . . . | 9 |
| Improved . . . . . . . . . . . . . . . . . | 16 |
| Relieved . . . . . . . . . . . . . . . . | 1 |
| Discharged by request of friends . . . . . . . . | 14 |
| Eloped . . . . . . . . . . . . . . . . . | 2 |

but, after a brief absence, were re-admitted, and finally discharged cured. These cases should be added to the cures in the former table."

Physicians to Institutions for the Insane are frequently questioned in reference to the time necessary to effect a restoration, in cases of insanity. The following table shows the term of residence in the Asylum, of all the patients who were discharged cured on their first admission:—

| | Males. | Females. | Total. |
|---|---|---|---|
| Less than one month . . . . | 45 | 31 | 76 |
| From one to two months . . . | 76 | 37 | 113 |
| From two to three months . . | 66 | 41 | 107 |
| From three to four months . . | 61 | 41 | 102 |
| From four to five months . . . | 29 | 25 | 54 |
| From five to six months . . . | 25 | 20 | 45 |
| From six to seven months. . . | 29 | 17 | 46 |
| From seven to eight months . . | 10 | 12 | 22 |
| From eight to nine months . . | 8 | 6 | 14 |
| From nine to ten months . . . | 13 | 5 | 18 |
| From ten to eleven months . . | 5 | 7 | 12 |
| From eleven to twelve months . | 11 | 4 | 15 |
| From one to two years. . . . | 23 | 12 | 35 |
| Upwards of two years . . . . . | 7 | 6 | 13 |

The whole number who were in the Asylum less than three months each, is 296. This is equivalent to 44 in every 100 that were cured. The whole number from three to six months is 201, or 30 in every hundred. The whole number from six to twelve months is 127, nearly 19 in every 100.

The whole number who were here upwards of one year each, is 48, or seven in every 100.

The mean or average term of residence in the Asylum was, for the men, four months and 27 days; and for the women, five months and 26 days.

Setting aside the 13 cases in which the persons were here more than two years each, the average time will be, for men, four months and 10 days, and, for women, four months and 25 days.

The mean or average time of residence of both sexes, inclusive, is five months and eight days. Excluding the 13 cases before mentioned, it is four months and 16 days.

Many people who take their friends to an institution of this kind, appear to be impressed with the idea that, if a restoration be possible it can be effected in a few days, as in an ordinary fever. But insanity, particularly in those cases which are sufficiently prolonged to induce their friends to remove them to an asylum, is essentially a chronic disease, and, even under the most skilful management, requires a considerable time for its removal, and the establishment of mental health. Were it possible always to induce the friends and guardians of patients to leave them at the asylum a sufficient time, fully and satisfactorily to test their curability by the restorative means here employed, the recoveries would undoubtedly be augmented, and that to no small extent.

The subjoined table establishes that by far the greatest number of cures was in persons between 20 and 30 years of age.

| | Males. | | Females. | | Total. |
|---|---|---|---|---|---|
| Under twenty years . . . | 30 | ... | 30 | ... | 60 |
| From twenty to thirty years . | 158 | ... | 113 | ... | 271 |
| From thirty to forty years . | 107 | ... | 55 | ... | 162 |
| From forty to fifty years . . | 57 | ... | 29 | ... | 86 |
| From fifty to sixty years . . | 25 | ... | 19 | ... | 44 |
| From sixty to seventy years . | 11 | ... | 2 | ... | 13 |
| From seventy to eighty years | 3 | ... | 0 | ... | 3 |
| From eighty to ninety years . | 0 | ... | 1 | ... | 1 |
| Total . . . | 391 | ... | 249 | ... | 640 |

"It will, however, be recollected that the number of patients who, at the time of admission, were between 20 and 30, very much exceeded that of those who were in any other decennium of life. Consequently, although the actual number of cures at that age greatly predominates, it does not necessarily follow that a larger *proportion* recovered than in some of the other periods."

Of the whole number of patients of different ages, the proportion of cures was greatest in those who were under 20; and that proportion diminishes, progressively and regularly, through all the subsequent decades of human life, as far as the eightieth year. There were but three patients above 80 years of age, and one of them was cured. This is equivalent to 33 and one-third per cent.; but the number is so small, as to be of no value in establishing the proportion of cures at that period of life, and altogether insufficient to unsettle the general rule apparent in the other cases, of diminishing curability in the advancing stages of life.

If the subject be investigated in regard to the sexes, separately, it will be found that the greatest proportion of cures was under 20 years of age; and that the progressive diminution of that proportion in the higher decades of life, holds good, with but a single exception, in either sex.

Of the men, about one per cent. more were cured in the age from 60 to 70 years than in that from 50 to 60; and in the women, the cures between 50 and 60 years were somewhat more than three per cent. greater than those between 40 and 50.

The proportion of cures among women under 20 years of age was more than 12½ per cent. greater than that of men; but in all the other periods, the proportion of men predominated over that of women, with the exception of the period from 50 to 60 years, when there was but very little difference in the two sexes.

The decennium from 80 to 90, being so palpably exceptionable, is not taken into account.

Hence, the old doctrine that mental alienation is more curable in early life than at more advanced periods, is strongly supported by these statistics.

The following table gives the causes of death, or of the diseases which terminate fatally, so far as they are recorded.

1*st. Deaths on first Admission.*

| | | | |
|---|---|---|---|
| Apoplexy, congestion of brain . . . . . . | 15 | Disease of heart . . . | 1 |
| Asthenia, atrophy . . . | 16 | Epilepsy . . . . . . | 9 |
| Abscess, lumbar . . . | 1 | Erysipelas . . . . . | 1 |
| Abscess of liver . . . | 1 | Fever, typhoid . . . . | 6 |
| Concussion of brain . . | 2 | „ bilious remittent . | 1 |
| Convulsions, epileptiform | 4 | „ intermittent . . | 1 |
| Cholera-morbus . . . | 2 | „ scarlet . . . . | 1 |
| Cholera, Asiatic . . . | 2 | Inflammation of bowels . | 3 |
| Cancer . . . . . . . | 1 | Inflammation of brain . | 10 |
| Consumption . . . . | 21 | Inanition . . . . . | 3 |
| Dropsy . . . . . . . | 2 | Paralysis . . . . . | 19 |
| Diarrhœa . . . . . | 5 | Suicide . . . . . . | 4 |
| Dysentery . . . . . | 7 | Strangulation while eating (paralytic) . . . | 2 |

2*nd. Deaths on Re-admission.*

| | | | |
|---|---|---|---|
| Apoplexy . . . . . | 4 | Dysentery . . . . . | 1 |
| Asthenia, marasmus . . | 3 | Disease of heart . . . | 1 |
| Consumption . . . . | 1 | Epilepsy . . . . . . | 2 |
| Dropsy . . . . . . . | 1 | Paralysis . . . . . | 4 |
| Diarrhœa . . . . . | 2 | | |

In concluding our analytical review of the history of the Bloomingdale Asylum, we beg to express our satisfaction at the mode in which its history has been recorded. The work is written with care, and the statistical tables appear to be drawn up with great attention to accuracy. We very much regret that the volume does not contain some information relative to the medical treatment of insanity.

